# Decoupling analysis of economic development and human well-being: A case study of the Qinghai-Xizang Plateau, China

**DOI:** 10.1371/journal.pone.0312041

**Published:** 2024-10-16

**Authors:** Weiguo Fan, Kehan Chen, Nan Chen, Meng Mengmeng, Xuechao Wang

**Affiliations:** 1 Department of Economic Management, North China Electric Power University, Baoding, China; 2 School of Management, Hebei University, Baoding, China; 3 School of Natural Resources, Faculty of Geographical Science, Beijing Normal University, Beijing, China; Tsinghua University, CHINA

## Abstract

Clarifying the relationship between economic development and human well-being is conducive to promoting high-quality economic development. This study focused on 16 prefecture-level cities in the Qinghai-Xizang Plateau region. The critic weighting method assessed the 2007–2018 human well-being index (HWI). The Tapio decoupling model allowed the study of the human well-being decoupling state. Finally, the drivers of decoupling between economic development and human well-being were analyzed using the Logarithmic Mean Divisia Index method. The results indicated that (1) almost all cities in the study region had an upward 2007–2018 HWI trend, but there were significant differences in growth magnitude and change trend. (2) Economic development and human well-being in the study region in 2007–2018 had expansion negative decoupling, thus, human well-being increased with economic growth, but not as fast as gross domestic product. 9 cities in 2007 showed weak decoupling, expansion connection, and expansion negative decoupling, increasing to 13 cities by 2018, indicating that human well-being development gradually improved from 2007 to 2018. (3) For most cities, the economic scale effect was the most influential factor in the decoupling of economic development and human well-being. Therefore, this study provided policy recommendations for decoupling economic development and human well-being.

## 1. Introduction

In recent years, quality of life has shown an upward trend, and the concept of human well-being has been mentioned by an increasing number of scholars and has become a hot topic. The multidimensional construct of human well-being is typically characterized by the interplay of health, happiness, and financial security. It is a holistic state that transcends mere existence, reflecting an individual’s profound contentment with their lifestyle, life circumstances, and aspirations [1, 2]. Well-being, as conceptualized in academic literature, is the embodiment of an individual’s subjective evaluation of their life, encapsulating a sense of fulfillment and satisfaction with one’s current status and pursuits. In other words, improving human well-being implies enhancing happiness and satisfaction. According to the Communist Party of China’s 20th National Congress report from 2022, “promote people’s well-being and improve their quality of life,” which indicates that human well-being is becoming increasingly important in daily life and has become one of the key tasks of China in recent years. The purpose of economic development is to improve human well-being, and the two are interrelated, interdependent, and affect each other. Therefore, improving human well-being while developing the economy has become an urgent issue for parties and countries. Generally speaking, the higher the material level, the happier people’s life is, thus, the more developed the economy is, the higher human well-being should be; however, related studies have shown that this is not necessarily the case [3]. Therefore, to determine how to improve human well-being while developing the economy, the connection between economic development and human well-being must first be clarified [4].

The Qinghai-Xizang Plateau, one of the world’s most distinctive geographical regions and a key site for implementing the Western Development Plan, is rich in natural resources [5]. However, because of the special geographical environment, complex human activities, and unreasonable industrial structure, the Qinghai-Xizang Plateau region faces numerous, intricate challenges related to social and economic growth, and there are significant development risks, which severely impede the Qinghai-Xizang Plateau region’s excellent regional growth [6]. The evaluation of high-quality regional development has become increasingly concerned with enhancing human well-being as the idea of development has evolved. In this regard, based on the Qinghai-Xizang Plateau region’s distinct geographic advantages, the development mindset should be altered, energy utilization efficiency should be increased, the economic development mode should be modified, the construction of economic infrastructure should be encouraged, and the intricate mechanism of economic well-being interactions in the region should be clarified by category. The Qinghai-Xizang Plateau region’s implementation of the requirements of high-quality development and the precise and comprehensive improvement of people’s well-being in the region depend greatly on identifying the essential components for improving human well-being and encouraging the continuous improvement of people’s living standards.

## 2. Literature review

As society progresses, human well-being becomes the core of sustainable development, and economic development is the only means to achieve it, as an increasing number of studies have found [7]. Consequently, there is a growing body of research on human well-being that focuses on the following areas: for the study of the HWI, based on annual data from 2005 to 2018, Yin et al examined the relationship between subjective well-being and the Human Well-being Index in more than 150 countries [8]. Yee et al. examined the potential value of ecosystem restoration or resource management by combining indicators of human well-being with interpolation methods that overcome data availability constraints [9]. The Gallup-Sharecare Well-Being Index was utilized by Anita et al. to determine the degree of well-being in low-income countries [10]. Salti et al. proposed a youth well-being index focusing on youth for the first time and extending the measure of national well-being to non-nationals [11]. Chen et al. evaluated counties in the Sanjiangyuan region by constructing an HWI to evaluate the spatial and temporal distribution of people’s well-being and found that the HWI in Sanjiangyuan steadily increased [12].

Another area of study is that of the factors that influence human well-being. De Neve and Sachs analyzed the sustainable development goals by examining how each relates to well-being and found that in most cases, there is a strong positive correlation between them [13]. Li and Zhou conducted an analysis on the effects of pollution on human welfare and found that pollution has an indirect influence on health. They did this by using data on air, water, and solid waste pollution as well as cross-sectional data from the 2016 Chinese Household Tracking Survey [14]. Tariq and Xu’s analysis of the consequences of fossil energy usage and greenhouse gas emissions on human well-being and income demonstrated that green energy had a favorable influence on both. On the other hand, there was a negative impact on well-being and a positive impact on income [15]. Using factor analysis, When Yang et al. looked into how public health investments affected citizens’ subjective well-being and how geographical variations affected it, they found that public health investments had a large and positive influence. There was also found to be an inverse U-shaped association between the subjective well-being of the locals and the regional differences in public health investments [16].

The decoupling theory, which has gained prominence recently, was first proposed by the Organization for Economic Cooperation and Development to investigate the connection between resource use, economic growth, and environmental degradation. At the national scale, Vogel and Hickel developed and implemented a new method to assess whether the decoupling achievements of high-income countries are consistent with the Paris climate and equality goals, and the study found that 11 high-income countries achieved absolute decoupling between 2013 and 2019 [17]. Wang and Su investigated the relationship between economic growth and carbon emissions in 192 countries and found that there was a clear decoupling state in developing countries, but the decoupling state in developed countries was mainly concentrated in a stable weak decoupling state that transitioned to a strong decoupling state. Among these, energy intensity had the most evidently positive effect on the decoupling process [18]. In their analysis of the relationships between the energy economies of major global regions and nations, Guo et al. also looked into the possibility of decoupling and discovered that, while energy use and economic growth have been rising simultaneously in developing economies, they show a typical inverted U-shaped decoupling relationship in industrialized nations [19]. The decoupling status of developing countries was found to be inferior to that of developed countries, where the main factors driving the growth of energy consumption were the scale of research and development (R&D) and the effects of urbanization, and the main factors impeding it were technological advancements and urbanization. Wang and Zhang employed the Tapio model to assess the decoupling status between energy consumption and the economy in five major energy-consuming countries [20]. At the provincial scale, the decoupling relationship between economic and indicators related to the electricity industry was analyzed at the national and provincial levels by Li et al. using the Tapio decoupling technique. They discovered that the decoupling relationship between economic and CO2 emissions in 30 Chinese provinces has regional characteristics and that national and international policies have an impact on the decoupling state [21]. Using the Tapio decoupling model, Gao et al. examined the province-level decoupling status of carbon emissions from economic development. They discovered that most provinces have weak decoupling between carbon emissions and economic development, with capital investment and total factor productivity serving as the main drivers of carbon emissions [22]. In their investigation of the decoupling effect of transport CO2 emissions in Chinese provinces, Cai et al. used the Tapio decoupling model and discovered that population scale, transport energy intensity, and per capita GDP all had an impact on the emissions’ rise [23]. Gan et al. examined the relationship between carbon emissions from the service sector and economic development in 30 Chinese provinces using the Tapio decoupling model. They discovered that, for the majority of the provinces under study, the relationship was only weakly decoupled [24].

Previous reports provide a good basis for the present study, but there are the following limitations. Firstly, most studies related to human well-being focus on the HWI and the influencing factors of human well-being, while ignoring the relationship between human well-being and economic development. Secondly, most decoupling studies have been conducted at the national and provincial scales, whereas studies at the municipal level are lacking. When studies are conducted at national and provincial scales, the accuracy of conclusions and the validity of recommendations are reduced. This study provides innovative insights to fill these two gaps. First, 16 typical prefecture-level cities in the Qinghai-Xizang Plateau region were selected as research areas. The economy of the Qinghai-Xizang Plateau region is relatively backward, and conducting research at the municipal level is conducive to formulating targeted policy recommendations for high-quality development in this region. Second, the decoupling relationship and drivers of economic development and human well-being were investigated using the Logarithmic Mean Divisia Index (LMDI) and Tapio decoupling models, which is helpful for the synergistic development of human well-being and the economy.

## 3. Methodologies and data sources

### 3.1 Data source

The present study takes into consideration the classification of human well-being elements based on the Millennium Ecosystem Assessment, the industrial structure of the farming-dependent Qinghai-Xizang Plateau region, the unique natural advantages of the region, the availability of data, and the definition of human well-being in terms of income and consumption (IAC), means of production (MOP), means of subsistence (MOS), and resource acquisition capability (RAC) in four dimensions to construct a human well-being indicator system [25–27]. The paper builds on previous research and examines the decoupling state and the drivers of decoupling. Among them, the IAC measures people’s living standards in four dimensions: income structure, consumer price, total income, and consumption structure; MOP measures the resources or tools that workers need to use for production in three dimensions: agricultural water, agricultural chemicals, and pasture; MOS measures the material conditions for human survival and development in three dimensions: agricultural products, livestock products, and means of travel; and RAC measures roads, operations, and the quality of people’s life services in three aspects: roads, operations, and telephones. Therefore, in this study, 18 corresponding assessment indicators were selected to construct an HWI system, and the established indicator system is shown in Table 1.

**Table 1:** HWI index system.

Category		Elements of human well-being	Evaluation index
Human well-being (HW)	Income and consumption (IAC)	Structure of income	Proportion of tertiary section (%)
		Consumption price	Consumer price index
		Total revenue	Urban per capita disposable income (Yuan)
			Rural per capita disposable income (Yuan)
		Structure of consumption	Coefficient of engel
	Means of production (MOP)	Agricultural water	Agricultural irrigation water rate (%)
		Agricultural chemistry	Fertilizer input per unit area (ton/ha)
			Pesticide input per unit area (ton/ha)
		Pasture	Per capita grassland area (Ha/person)
	Means of subsistence (MOS)	Agricultural products	Per capita grain consumption (ton/person)
			Per capita vegetable consumption (ton/person)
		Animal products	Per capita meat consumption (ton/person)
			Per capita quantity of eggs (ton/person)
		Travel tool	Per capita ownership of cars and motorcycles (units / 100 households)
	Resource acquisition capability (RAC)	Road transport	Road freight turnover (tons ∙ km)
		Transportation	Passenger traffic (ten thousand)
			Freight volume (10,000 tons)
		Telephone	Landline subscribers (10,000 households)

The China Science and Technology Statistical Bulletin and the China Statistical Yearbook provided the data for this investigation. The data on research and experimental expenditures were obtained from the China Science and Technology Statistical Bulletin from 2008 to 2019, and the remaining 22 data points, such as proportion of tertiary section, consumer price index, and urban per capita disposable income, were obtained from the China Statistical Yearbook from 2008 to 2019. Meanwhile, to exclude the effect of inflation, data on regional GDP, fixed asset investment, and research and experimental expenditure were adjusted to constant 2007 prices.

### 3.2 LMDI method

The main methods used for the analysis of decoupling influence factors were factor decomposition, coefficient analysis, the equation method, and the causality method. Compared to other methods, the factor decomposition method was easy to calculate and required less application for data size. Among them, the LMDI method is the most widely used [28, 29]. Therefore, this study used the LMDI method to decompose the driving factors of decoupling in the Qinghai-Xizang Plateau. The model is expressed as follows:

$$Y=\sum_{ij}Y_{ij}=\sum_{ij}\left(\frac{Y_{ij}}{Y_{i}}\times \frac{Y_{i}}{R_{i}}\times \frac{R_{i}}{I_{i}}\times \frac{I_{i}}{Y_{i}}\times \frac{Y_{i}}{P_{i}}\times P_{i}\right)$$

$$=\sum_{i}\left(M\times T\times N\times S\times Q\times U\right)$$ (1)

In Eq (1), Y is the GDP, *Y*_*i*_ is the GDP of *i* city, *Y*_*ij*_ is the GDP of *j* industry in *i* city, *R*_*i*_ is the experimental and research funds of *i* City, *I*_*i*_ is the investment in fixed assets of *i* city, *P*_*i*_ is the population of the city $i,M=\frac{Y_{ij}}{Y_{i}}$ is the factor of industrial structure, $T=\frac{Y_{i}}{R_{i}}$ is the factor of R&D efficiency, $N=\frac{R_{i}}{I_{i}}$ is the factor of R&D intensity, $S=\frac{I_{i}}{Y_{i}}$ is the factor of investment intensity, $Q=\frac{Y_{i}}{P_{i}}$ is the factor of economic scale, and *U* = *P*_*i*_ is population size.

Therefore, the LMDI additive decomposition method could be used to decompose the change in economic development into the following effects:

$$\Delta Y=Y^{t}-Y^{0}=\sum_{t}\left({M^{t}\times T}^{t}\times N^{t}\times S^{t}\times Q^{t}\times U^{t}\right)-\sum_{t}\left({M^{0}\times T}^{0}\times N^{0}\times S^{0}\times Q^{0}\times U^{0}\right)$$

=ΔM+ΔT+ΔN+ΔS+ΔQ+ΔU (2)

In Eq (2), Δ*M* is the industry structure effect, Δ*M* is the R&D efficiency effect, Δ*N* is the R&D intensity effect, Δ*S* is the investment intensity effect, Δ*Q* is the economic scale effect, and Δ*U* is the population size effect.

Coefficients in the LMDI model were calculated as follows:

$$W_{ij}=\frac{Y_{ij}^{t}-Y_{ij}^{0}}{lnY_{ij}^{t}-lnY_{ij}^{0}}$$ (3)

In Eq (3), $Y_{ij}^{t}$ denotes the GDP of industry *j* in city *i* in year *t*, and $Y_{ij}^{0}$ denotes the GDP of industry *j* in city *i* in the base year. Then, the effects of each factor on economic development are expressed as follows:

$$\Delta M=\sum_{ij}W_{ij}\times ln\frac{F^{t}}{F^{0}}$$ (4)

$$\Delta T=\sum_{ij}W_{ij}\times ln\frac{T^{t}}{T^{0}}$$ (5)

$$\Delta N=\sum_{ij}W_{ij}\times ln\frac{N^{t}}{N^{0}}$$ (6)

$$\Delta S=\sum_{ij}W_{ij}\times ln\frac{S^{t}}{S^{0}}$$ (7)

$$\Delta Q=\sum_{ij}W_{ij}\times ln\frac{Q^{t}}{Q^{0}}$$ (8)

$$\Delta U=\sum_{ij}W_{ij}\times ln\frac{U^{t}}{U^{0}}$$ (9)

### 3.3 Tapio decoupling model

A decoupling model of economic development and human well-being was constructed based on the LMDI factor decomposition method combined with the Tapio decoupling elasticity index [30]. The model was as follows:

$$D=\frac{\frac{\Delta Y}{Y^{0}}}{\frac{\Delta HW}{{HW}^{0}}}=\Delta Y\times \frac{{HW}^{0}}{Y^{0}\times \Delta HW}=\Delta M+\Delta T+\Delta N+\Delta S+\Delta Q+\Delta U)\times \frac{{HW}^{0}}{Y^{0}\times \Delta HW}$$

$$=D_{M}+D_{T}+D_{N}+D_{S}+D_{Q}+D_{U}$$ (10)

In Eq (10), *D* is the decoupling index of economic development and human well-being, Δ*Y* is the value-added of GDP, *Y*^0^ is the GDP in the base period, Δ*HW* is the value-added of human well-being, *HW*^0^ is the IAC dimensions of human well-being in the base period, *D*_*M*_ is the industrial structure effect, *D*_*T*_ is the R&D efficiency effect, *D*_*N*_ is the R&D intensity effect, *D*_*S*_ is the investment intensity effect, *D*_*Q*_ is the economy size effect, and *D*_*U*_ is the population size effect. Table 2 presents the decoupling states and their classification standards.

**Table 2:** State of decoupling and classification standard.

Decoupling status	Δ*Y*	Δ*HW*	*D*
Weak decoupling (WD)	≥0	≥0	0≤D≤0.8
Strong decoupling (SD)	<0	>0	D<0
Recession decoupling (RD)	<0	<0	D>1.2
Expansion negative decoupling (END)	>0	>0	D>1.2
Strong negative decoupling (SND)	>0	<0	D<0
Weak negative decoupling (WND)	≤0	≤0	0≤D≤0.8
Expansion connection (EC)	>0	>0	0.8≤D≤1.2
Weak connection (WC)	<0	<0	0.8≤D≤1.2

## 4. Results

### 4.1 Analysis of human well-being in the Qinghai-Xizang Plateau region

**Fig 1:**
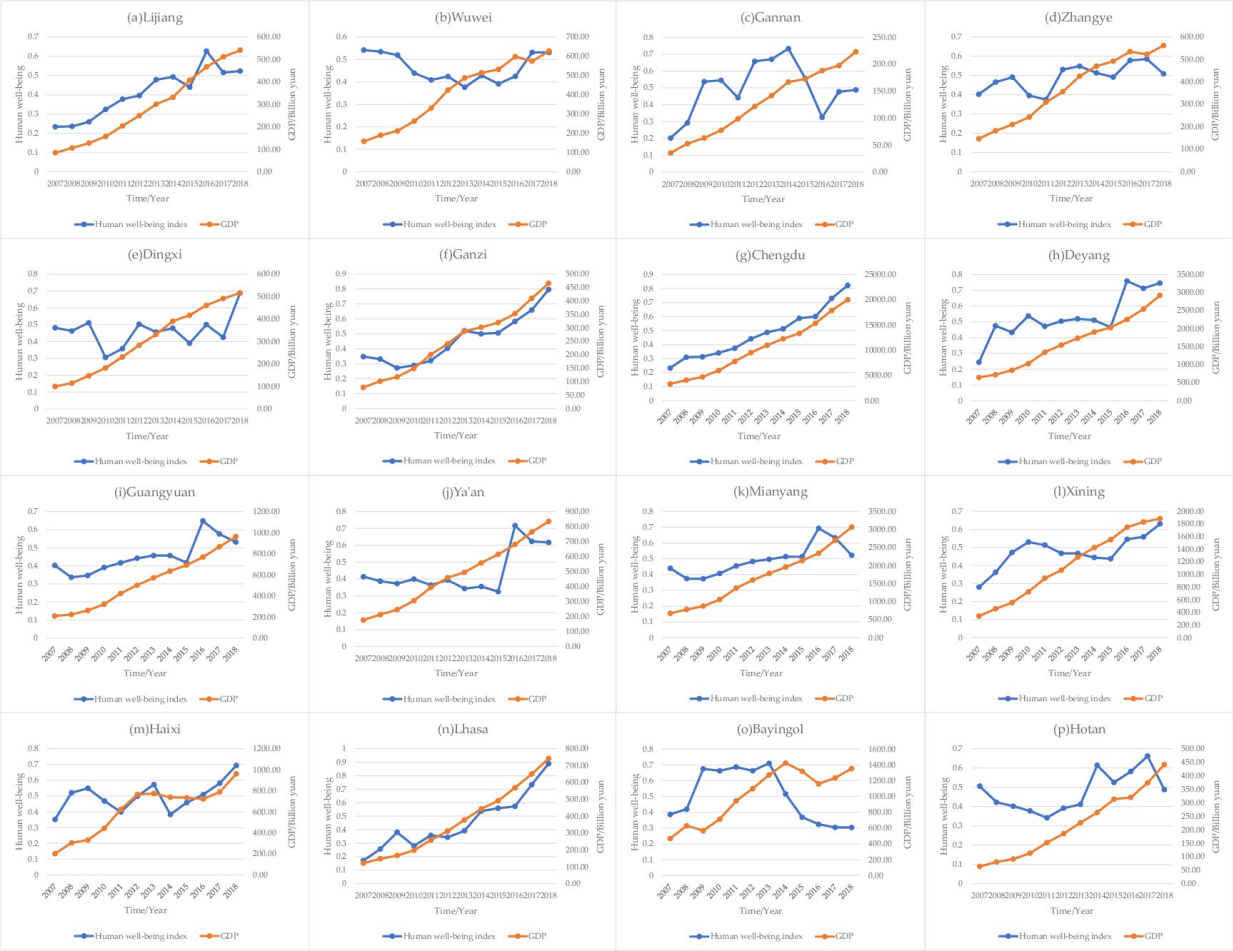
Trends in HWI and GDP in the Qinghai-Xizang Plateau region, 2007–2018.

First, the HWI was obtained by synthesizing the selected 18 objective indicators after dimensionless processing using the critic weighting method. In terms of the trend of changes in human well-being in the Qinghai-Xizang Plateau region, the human well-being and economic levels of 16 typical prefecture-level cities showed an overall growth trend (Fig 1), which agrees with Tian et al.’s conclusions [31]. Overall, the Qinghai-Xizang Plateau region’s 13 prefecture-level cities’ HWI, except for Wuwei, Bayingol, and Hotan, increased in 2018 compared to 2007, but the magnitude of growth as well as the trend of change differed significantly. Cities can be divided into three categories based on differences in changes in human well-being in the Qinghai-Xizang Plateau region (Table 3).

**Table 3:** Growth of HWI by cities in the Qinghai-Xizang Plateau region, 2007–2018.

Category	Growth rate	City
Ⅰ	≥100%	Lijiang, Ganan, Ganzi, Chengdu, Deyang, Xining, Lhasa
Ⅱ	(0,100%]	Zhangye, Dingxi, Guangyuan, Ya’an, Mianyang, Haixi,
Ⅲ	≤0	Wuwei, Bayingol, Hotan

The overall growth of the HWI in Category I cities was large, but there were differences in the changes in different cities. Lijiang’s HWI fluctuated and peaked at 0.63 in 2016, representing an increase of 167.8% from 2007 (Fig 1A). The HWI of Gannan showed an upward trend and peaked at 0.73 in 2014 but then began to decline significantly and started to rise again in 2016 (Fig 1C). The HWI of Ganzi fluctuated but showed an overall increasing trend, increasing by 129% in 2018 compared with 2007 (Fig 1F). The HWI in Chengdu continued to increase at an average rate of 23% per year (Fig 1G). The HWI of Deyang showed substantial increases in 2008 and 2016, with little change in the other years (Fig 1H). The HWI of Xining continued to grow from 2007–2010, slowly declined from 2011–2015, and started to grow again in 2016, increasing by 125% in 2018 compared with 2007 (Fig 1L). The HWI of Lhasa fluctuated but generally showed growth, increasing by 419.7% in 2018 compared with 2007 (Fig 1N).

The magnitude of the HWI change in Class II cities was relatively small, but the trend varied widely among cities. The HWI of Zhangye showed a large variation between 2007–2018 but an overall M-shaped variation, with an increase of 26.3% in 2018 compared to 2007 (Fig 1D). The HWI of Dingxi showed fluctuations but little overall change from 2007–2017, and suddenly increased sharply in 2018 by 42.8% compared to in 2007 (Fig 1E). The HWI of Guangyuan, Ya’an, and Mianyang fluctuated but changed little from 2007–2015 and suddenly increased in 2016, and then began to decline (Fig 1I–1L). The HWI of Haixi showed an M-shaped trend from 2007–2014 and started to increase continuously in 2015, and increased to 97.1% in 2018 compared with 2007 (Fig 1M).

The HWI of Class III cities decreased, but this decrease was not statistically significant. The HWI of Wuwei decreased annually to a minimum of 0.38 in 2013, a decrease of 30.5% compared with 2007, and then began to gradually increase in 2018 to almost the same level as in 2007 (Fig 1B). The HWI of Bayingol showed a significant increase of 60.7% in 2009 compared with the previous year, followed by a small fluctuation and a yearly decrease from 2014 (Fig 1O). Hotan’s HWI showed a decreasing trend from 2007 to 2011 and an M-shaped trend after 2012 (Fig 1P).

### 4.2 Analysis of the decoupling of economic growth and human well-being

#### 4.2.1 Analysis of the state of decoupling between economic growth and human well-being

This study, which used the Tapio model as its foundation, looked at the decoupling relationship between economic growth and human well-being in 16 typical Qinghai-Xizang Plateau cities between 2007 and 2018. The findings are displayed in Table 4.

**Table 4:** Decoupling status of economic development and human well-being by cities in the Qinghai-Xizang Plateau region, 2007–2018.

	2007–2008	2008–2009	2009–2010	2010–2011	2011–2012	2012–2013	2013–2014	2014–2015	2015–2016	2016–2017	2017–2018
Lijiang	END	END	END	END	END	END	END	END	END	END	END
Wuwei	SND	SND	SND	SND	SND	SND	SND	SND	SND	SND	SND
Gannan	EC	WD	WD	END	EC	END	END	END	END	END	END
Zhangye	END	END	SND	END	END	END	END	END	END	EC	END
Dingxi	SND	END	SND	SND	END	SND	SND	SND	END	SND	END
Ganzi	SND	SND	SND	SND	END	END	END	END	END	END	END
Chengdu	WD	END	END	END	END	END	END	END	END	END	END
Deyang	WD	WD	WD	WD	END	END	END	END	EC	END	END
Guangyuan	SND	SND	SND	END	END	END	END	END	END	END	END
Ya’an	SND	SND	SND	SND	SND	SND	SND	SND	END	END	END
Mianyang	SND	SND	SND	END	END	END	END	END	END	END	END
Xining	EC	EC	END	END	END	END	END	END	END	END	END
Haixi	EC	EC	END	END	END	END	END	END	END	END	END
Lhasa	WD	WD	EC	EC	END	END	END	END	END	END	END
Bayingol	END	WD	WD	END	END	END	END	SND	END	SND	SND
Hotan	SND	SND	SND	SND	SND	SND	END	END	END	END	SND

The Qinghai-Xizang Plateau’s overall decoupling connection between economic growth and human well-being was evidently in an expansion negative state between 2007 and 2018, meaning that HWI increased with economic growth but did so more slowly. Economic growth and human well-being in Lijiang showed expansion-negative decoupling during 2007–2018. Wuwei showed SND between economic growth and human well-being, implying that the level of human well-being decreases with economic development. Gannan showed EC-WD-END, indicating that the level of human well-being increases continuously with economic growth, during which the growth rate of human well-being exceeds that of economic development, but then slows down. Zhangye briefly showed SND and EC; however, overall, it showed END. Dingxi alternated between SND and END, indicating that its level of human well-being responded erratically to economic growth. Ganzi showed SND from 2007–2011 and END from 2011–2018, indicating that the level of human well-being first decreased and then increased with economic growth. Chengdu showed overall END, indicating that the level of human well-being increased with economic growth, but at a slower rate than economic growth. Deyang shows a WD-END change, indicating that its human well-being level first increased faster than economic growth, but then slower than economic growth. Guangyuan and Mianyang changed similarly, both showing SND between 2007 and 2009 and END from 2010 onwards. Ya’an showed SND from 2007–2015, but showed END from 2016 onwards. Both Xining and Haixi showed an EC-END trend. Lhasa exhibited an EC-WD-END trend during 2007–2018, while Bayingkol exhibited a WD-END-SND change scenario. Hotan showed SND from 2007–2013, but END from 2014 onwards.

Nine cities in the Qinghai-Xizang Plateau region showed WD, WC, and END in 2007–2008, and this number increased from nine to 13 in 2008–2018, with most cities experiencing economic development favorable to human well-being. Gannan, Deyang, and Lhasa reached a WD state in some years, but their stability was poor. Meanwhile, some cities, such as Guangyuan and Mianyang, experienced a SND phase before reaching an END state. A small number of cities, including Dingxi, repeatedly experienced SND and END but gradually showed an END status. Wuwei, on the other hand, is in a SND state. In general, most cities reached an END state near the end of the study period, but the decoupling evolution trends were relatively unstable.

#### 4.2.2 Analysis of the drivers of decoupling economic growth and human well-being

**Fig 2:**
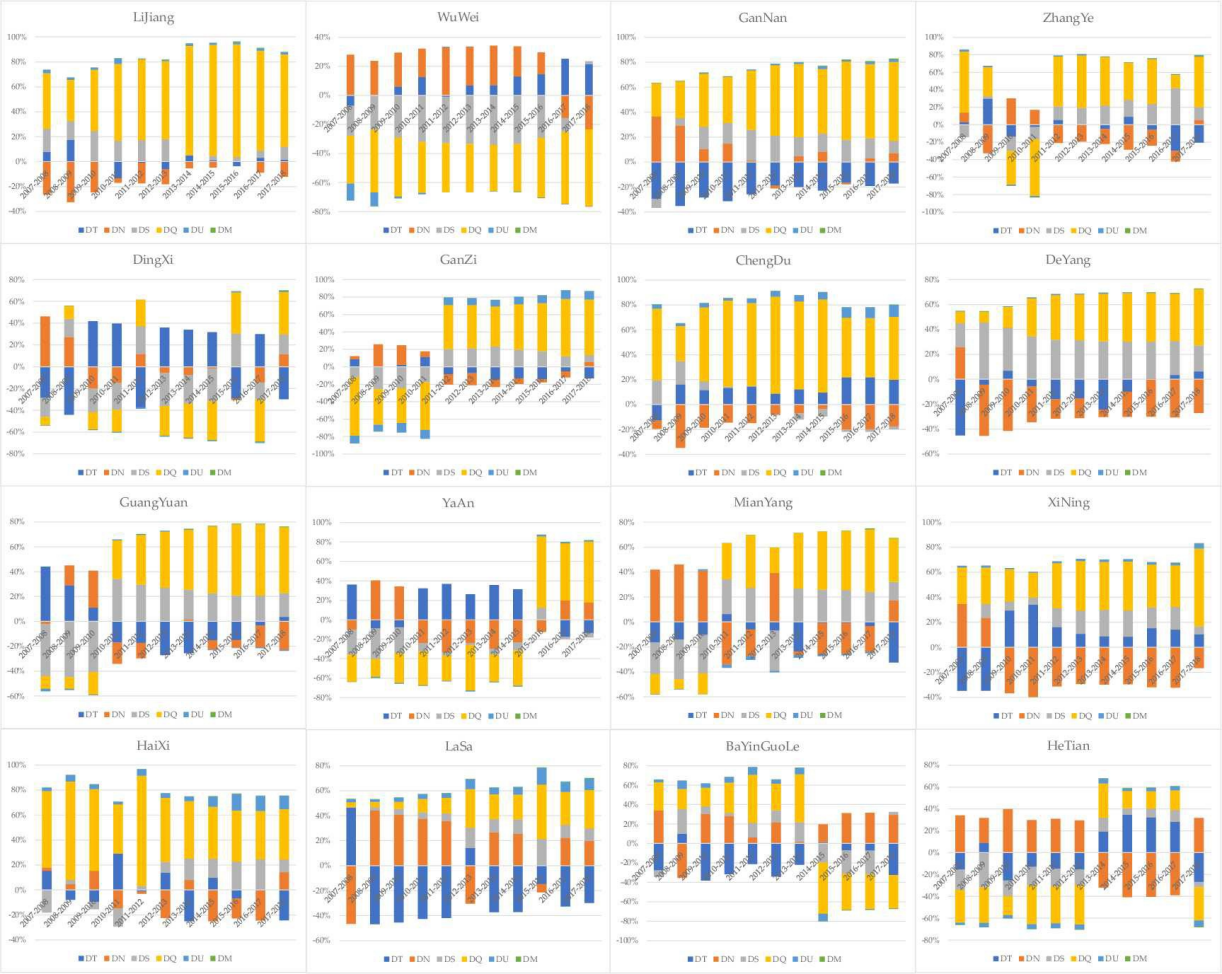
Contribution of decoupling driving effects of economic development and human well-being in cities in the Qinghai-Xizang Plateau region, 2007–2018.

The decoupling state can only describe the synchronous relationship between economic development and human well-being and cannot explain the mechanism of changes in human well-being. Therefore, the decomposition of the decoupling drivers of economic development and human well-being for each typical city in the Qinghai-Xizang Plateau region from 2007–2018 based on the LMDI model yields the contributions of the drivers of R&D efficiency, R&D intensity, investment intensity, economic scale, population size, and industrial structure, as shown in Fig 2 and Table 6. The classification of cities according to the largest hook drivers affecting decoupling is shown in Table 5.

**Table 5:** Largest influences on the decoupling from 2007–2018.

	Economic scale effect	R&D efficiency effect	R&D intensity effect
City	Lijiang, Wuwei, Gannan, Zhangye, Ganzi, Chengdu, Deyang, Guangyuan, Ya’an, Mianyang, Xining, Haixi, Bayingol	Dingxi, Lhasa	Hotan

According to the chart, it can be seen that the R&D intensity effect pair is the main factor driving decoupling in Hotan, while in Dingxi and Lhasa the R&D efficiency effect plays a dominant role, and in the remaining cities the economic scale effect has the greatest impact on decoupling economic development from human well-being. This may be because Hotan, Dingxi and Lhasa all attach great importance to the development of science and technology, but Hotan’s time and speed of promoting scientific and technological innovation are still slower than Dingxi and Lhasa’s. During the research period, although R&D investment is more, R&D output is not high. For most cities, it has been gradually realized that the purpose of development is to pursue a happy life, and GDP is no longer the only criterion to measure social well-being and economic structure change. Social development and progress should be manifested not only in the increase of social wealth, but also in the enhancement of human subjectivity and initiative, the expansion of freedom and rights, the improvement of the overall quality of life, and the free and all-round development of human beings. Although the economic scale effect plays a dominant role in 13 cities, there are also large differences across cities. From the perspective of all kinds of cities, except Ganzi, the economic scale effect of Class I cities has promoted decoupling; In Class II and III cities, during the research period, only the economic scale effect of Haixi completely promoted the decoupling, and the economic scale effect of Wuwei completely inhibited the decoupling. In other cities, the economic scale effect basically had the opposite effect on decoupling in the early and late research periods. For most cities in the Qinghai-Xizang Plateau region, the economic scale effect is an important influencing factor in the decoupling of economic development and human well-being, and even the economic scale effect in some cities exceeds 50% and is a decisive factor in decoupling. This also shows that the economic aggregate is important, but excessive consumption of environment and resources to improve GDP is only applicable to the early stage of economic growth with the main purpose of economic aggregate growth, not to the late stage when all-round development has been paid attention to, and even less to the implementation of Scientific Outlook on Development. No longer only pay attention to the enhancement of economic strength, it will undoubtedly be used to better solve the problem of harmonious development of domestic society, and improving national happiness is the greatest value of GDP. Besides the difference of human well-being index, other factors will also cause some differences between cities, so other driving factors have different effects on decoupling except the effect of economic scale. The investment intensity effect has the same direction of influence as the economic scale effect in most of the regions, but the effect is not as effective as the economic scale effect. This also proves to some extent that investment and economic growth are intrinsically and inevitably related, and they restrict and promote each other. The effects of R&D efficiency and R&D intensity vary and are unstable for different cities, and are less influential than the economic scale effect in most regions. This may be because the R&D input of different industries in different cities has different effects on regional innovation output, and the R&D input of traditional industries and high-tech industries has different effects on regional innovation output. In leading areas and catching-up areas, the R&D investment of traditional industries contributes to regional industrial innovation, while the R&D investment of high-tech industries has always promoted the innovation output of leading areas, but only after it has developed to a certain extent in catching-up areas. This also shows that it is very important to allocate R&D investment reasonably according to the different development stages and industrial structure of the region. ‌The population size effect has a small effect on decoupling, and the effect of industrial structure effect is almost negligible.

## 5. Discussion

### 5.1 Analysis of the decoupling status in different dimensions

In this study, the HWI was calculated by selecting 18 basic indicators and the decoupling relationship between economic growth and human well-being at the municipal level was studied using the LMDI decomposition and Tapio decoupling model. However, only the decoupling relationship between economic development and human well-being was considered, and we could not capture the impact of economic development on different aspects of human well-being. Therefore, subsequent studies will begin with the four dimensions of IAC, MOP, MOS, and RAC to investigate their decoupling status and the drivers of economic development.

Table 6 shows the main decoupling states of typical cities in the Qinghai-Xizang Plateau region in the four dimensions from 2007–2018. In the IAC dimension, Lijiang and Lhasa were mainly in a WD state between economic growth and human well-being during 2007–2018, indicating that the HWI grew faster with GDP and HWI. Hotan was mainly in a SD state during 2007–2018, indicating that the HWI decreased with GDP growth, whereas the remaining 13 cities were mainly in an expansion-negative decoupling state, indicating that the HWI grew with GDP growth, but the growth rate of the HWI was not as fast as that of GDP. In the MOP dimension, Deyang and Xining were mainly in a WD state; Chengdu, Haixi, and Bayingol were mainly in an expansion-negative decoupling state; and the remaining cities were mainly in a SND state. In the MOS dimension, Lhasa was mainly in WD state; Lijiang, Gannan, Zhangye, Chengdu, Deyang, Ya’an, Haixi, and Bayingol were mainly in an expansion-negative decoupling state during 2007–2018, and the remaining cities were mainly in a SND state. In the RAC dimension, Lijiang, Gannan, Chengdu, Guangyuan, and Lhasa were mainly in a WD state, Mianyang was mainly in a SND state from 2007–2018, and the remaining cities were mainly in an expansion-negative decoupling state. Overall, the decoupling of economic growth and human well-being under the IAC and RAC dimensions was good, and the HWI of most cities increased with GDP. The decoupling state under the MOP dimension was relatively poor and the HWI of most cities decreased with GDP growth, which is consistent with the conclusion of Ward et al [32].

**Table 6:** Major decoupling states in the Qinghai-Xizang Plateau region in the IAC, MOP, MOS, and RAC dimensions from 2007–2018.

	WD	END	SND
IAC	Lijiang, Lhasa	Wuwei, Gannan, Zhangye, Dingxi, Ganzi, Chengdu, Deyang, Guangyuan, Ya’an, Mianyang, Xining, Haixi, Bayingol	Hotan
MOP	Deyang, Xining	Chengdu, Haixi, Bayingol	Lijiang, Wuwei, Gannan, Zhangye, Dingxi, Ganzi, Guangyuan, Ya’an, Mianyang, Lhasa, Hotan
MOS	Lhasa	Lijiang, Gannan, Zhangye, Chengdu, Deyang, Ya’an, Haixi, Bayingol	Wuwei, Dingxi, Ganzi, Guangyuan, Mianyang, Xining, Hotan
RAC	Lijiang, Gannan, Chengdu, Guangyuan, Lhasa	Wuwei, Zhangye, Dingxi, Ganzi, Deyang, Ya’an, Xining, Haixi, Bayingol, Hotan	Mianyang

### 5.2 Analysis of the drivers of decoupling in different dimensions

Based on the analysis in Section 4.2.2, the economic scale effect was identified as the most important factor influencing the decoupling of economic development and human well-being, which is also consistent with the conclusion in the four dimensions of IAC, MOP, MOS, and RAC. Therefore, this study investigated the most influential decoupling factors under the four dimensions of IAC, MOP, MOS, and RAC (Table 7). The largest influencing factor affecting the decoupling of Hotan under the four dimensions of IAC, MOP, MOS, and RAA was the R&D intensity effect, that of Dingxi and Lhasa was the R&D efficiency effect, and that of the remaining 13 cities was the economic scale effect. The conclusions obtained under the four dimensions of IAC, MOP, MOS, and RAC were consistent with those obtained under the human well-being dimension in terms of decoupling drivers.

**Table 7:** Largest influences on the decoupling of the IAC, MOP, MOS, and RAC dimensions from 2007–2018.

	Economic scale effect	R&D efficiency effect	R&D intensity effect
IAC	Lijiang, Wuwei, Gannan, Zhangye, Ganzi, Chengdu, Deyang, Guangyuan, Ya’an, Mianyang, Xining, Haixi, Bayingol	Dingxi, Lhasa	Hotan
MOP	Lijiang, Wuwei, Gannan, Zhangye, Ganzi, Chengdu, Deyang, Guangyuan, Ya’an Mianyang, Xining, Haixi, Bayingol,	Dingxi, Lhasa	Hotan
MOS	Lijiang, Wuwei, Gannan, Zhangye, Ganzi, Chengdu, Deyang, Guangyuan, Ya’an, Mianyang, Xining, Haixi, Bayingol	Dingxi, Lhasa	Hotan
RAC	Lijiang, Wuwei, Ganan, Zhangye, Ganzi, Chengdu, Deyang, Guangyuan, Ya’an, Mianyang, Xining, Haixi, Bayingol	Dingxi, Lhasa	Hotan

### 5.3 Policy implication

The findings of this study also allowed the development of policy recommendations to improve human well-being and encourage the decoupling of economic development from human well-being.

1. Transforming economic development model. Municipalities in the Qinghai-Xizang Plateau region have experienced major improvements in their economic development since the turn of the century, yet the region still experiences uneven growth. The results of this study indicate that the economic scale is the main factor influencing the decoupling of economic development from human well-being, suggesting that the enhancement of human well-being relies on economic development. Therefore, cities in the Qinghai-Xizang Plateau region should actively transform their economic development patterns to enhance the role of economic scale in promoting decoupling and creating a favorable external environment for human well-being enhancement. For example, by developing the clean energy industry, Qinghai province has not only increased its economic scale, but also improved the quality of life of local residents and reduced environmental pollution [33].
2. Optimizing industrial structure. The results showed that the industrial structure had almost no effect on the decoupling of economic development from human well-being. In this regard, local governments should adjust and optimize the industrial structure in a timely manner, so that each industry can achieve coordinated development. By formulating reasonable policies and improving the supervision system, they can influence the change in industrial structure, realize the optimal allocation of resources, promote the rationalization and advanced development of industrial structure, and give full play to the positive effects of industrial structure. For example, through effective resource management policies, Norway has turned the rich oil resources in the North Sea into national wealth, and invested in education, research and infrastructure construction, achieving optimal allocation of resources and overall improvement of social well-being [34].
3. Emphasis on scientific research. Science and technology are the primary productive forces, and modern science and technology widely permeate economic activities and are determinants of economic development. The results of this study showed that R&D efficiency and intensity were important factors that influenced the decoupling of economic development and human well-being. In this regard, local governments should pay attention to scientific research, insist on R&D investment, enhance local competitive advantages, and facilitate scientific research so that it can play a positive role in driving local economic development and enhancing human well-being. For example, China’s high-speed railway technology has become a business card of China’s high-end manufacturing industry through continuous research and development investment, enhancing China’s competitiveness in the global infrastructure construction market [35].
4. Strengthening regional cooperation. The results of this study indicated that human well-being increases with economic development in most cities in the Qinghai-Xizang Plateau region; however, a small number of cities still exist (e.g., Wuwei, Bayingol, and Hotan) where human well-being decreases with economic development. Regions with poor decoupling status should learn from the development of regions with a better decoupling status (Zhao et al. 2022); organically organize dispersed economic activities by complementing, sharing, or superimposing advantages between regions; and stimulate potential economic vitality to promote the synergistic development of the economy and human well-being [36].

## 6. Conclusions

Based on the data from 16 cities, including Lijiang and Wuwei, from 2007–2018, this study calculated the HWI by establishing a human well-being indicator system and analyzed the decoupling relationship between economic development and human well-being and its driving factors using the LMDI and Tapio models. This research contributes to the implementation of high-quality development requirements, precise and comprehensive improvement of people’s livelihood, and continuous improvement of people’s living standards in the Qinghai-Xizang Plateau region. The main findings were as follows:

1. The HWI of the 13 prefecture-level cities in the Qinghai-Xizang Plateau region, except for Wuwei, Bayingol, and Hotan, increased in 2018 compared to 2007, but there were large differences in the magnitude of growth as well as in the trend of change.
2. Economic development and human well-being in the Qinghai-Xizang Plateau region as a whole showed negative decoupling from 2007–2018, indicating that human well-being increased with economic growth but not as fast as GDP. Nine cities showed WD, EC, and expansion-negative decoupling from 2007–2008, and this number increased from nine to 13 in 2018, indicating that most cities’ economic development was conducive to human well-being.
3. For most cities in the Qinghai-Xizang Plateau region, the economic scale effect was the largest influencing factor in the decoupling of economic development and human well-being, and even the economic scale effect in some cities had an impact of > 50% and was the decisive factor in decoupling. The investment intensity effect had the same direction of influence as the economic scale effect in most regions; however, the effect was not as effective as the economic scale effect. The effects of R&D efficiency and intensity varied, were unstable across cities, and were less influential than the economic-scale effect in most regions. The population size had a small effect on decoupling and the effect of the industrial structure was almost negligible.

However, this study had some limitations. Firstly, the critic method was used to synthesize the HWI from the bottom up, which may have led to bias in the calculation results. Secondly, due to the availability of data, the well-being system constructed in this paper only included objective well-being and lacked consideration of subjective economic well-being. Thirdly, this study only considered the impact of a city’s economy on its own well-being, ignoring interactions between cities.

## Supporting information

S1 Data (XLSX)
